# Medial Condyle Fracture (Kilfoyle Type III) of the Distal Humerus with Transient Fishtail Deformity after Surgery

**DOI:** 10.1155/2017/9053949

**Published:** 2017-12-14

**Authors:** Motoki Sonohata, Takema Nakashima, Hiroaki Suetsugi, Masaru Kitajima, Masaya Ueno, Masaaki Mawatari

**Affiliations:** Department of Orthopaedic Surgery, Faculty of Medicine, Saga University, 5-1-1 Nabeshima, Saga 849-8501, Japan

## Abstract

A “Fishtail deformity” is one of the well-known complications following pediatric lateral condyle or supracondylar fractures of the humerus. We herein report a case of medial condyle fracture (Kilfoyle type III) in an 11-year-old boy. He had a transient “fishtail deformity” of the trochlear groove after open reduction and internal fixation. As occurred in the current case, the bone remodeling and the improvement of ischemia of the trochlea after medial condyle fracture may be associated with the likelihood of recovery from transient “fishtail deformity.”

## 1. Introduction

Distal humerus fracture is very common in pediatric patients; however, fracture of the medial condyle of the humerus is very rare, accounting for only 1–2% of all pediatric elbow fractures [1–4]. Kilfoyle classified these fractures into three types according to the degree of displacement [5]. He achieved favorable results with open reduction and internal fixation, even in cases involving complete and displaced fractures (type III), by diagnosing them early. Some reports suggest that the outcomes of these fractures are uniformly poor when they are diagnosed late and treated with open reduction and internal fixation [1, 2, 6, 7]. Therefore, the delay in the diagnosis results in poor outcomes; however, the decision to treat via open reduction and internal fixation remains controversial. The clinical course of fracture of the medial condyle of the humerus is unclear. We herein report a case of medial condyle fracture (Kilfoyle type III) with transient fishtail deformity of the trochlear groove due to failure of the lateral trochlear ossification centers to develop after open reduction and internal fixation.

## 2. Case Report

**Figure 1:**
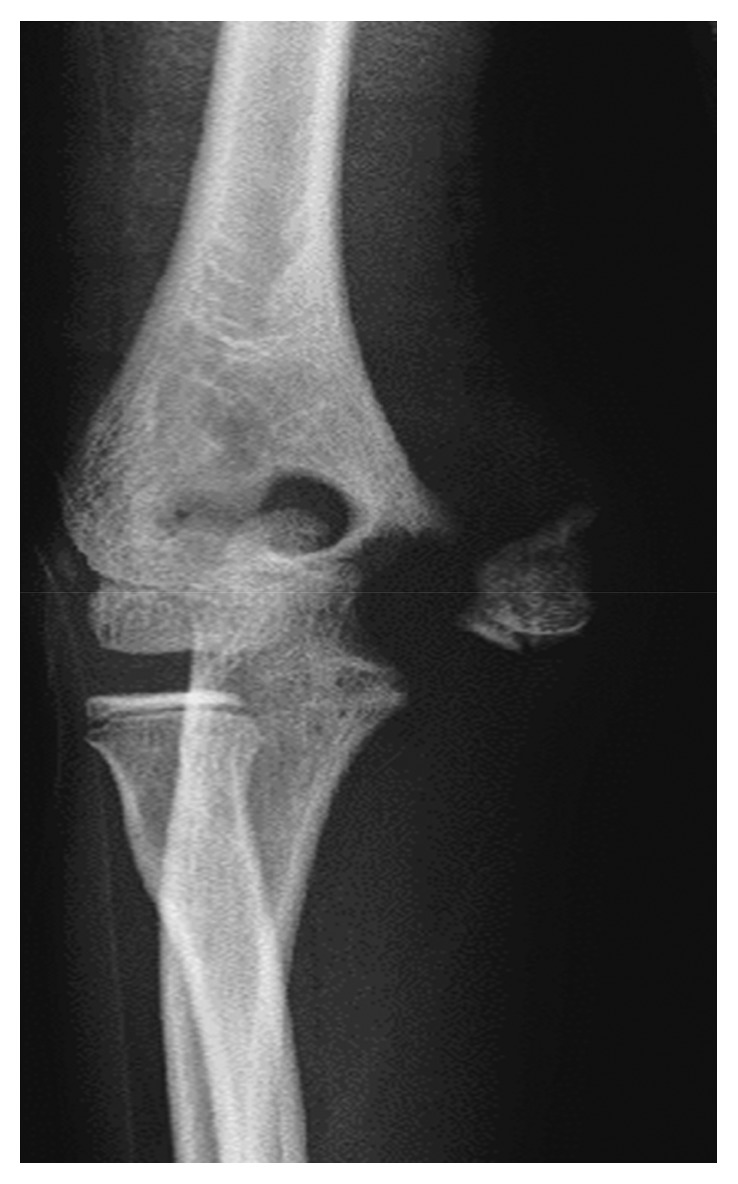
Anteroposterior radiographs of the right elbow at the initial visit. A medial condyle fracture (Kilfoyle type III) was seen.

**Figure 2:**
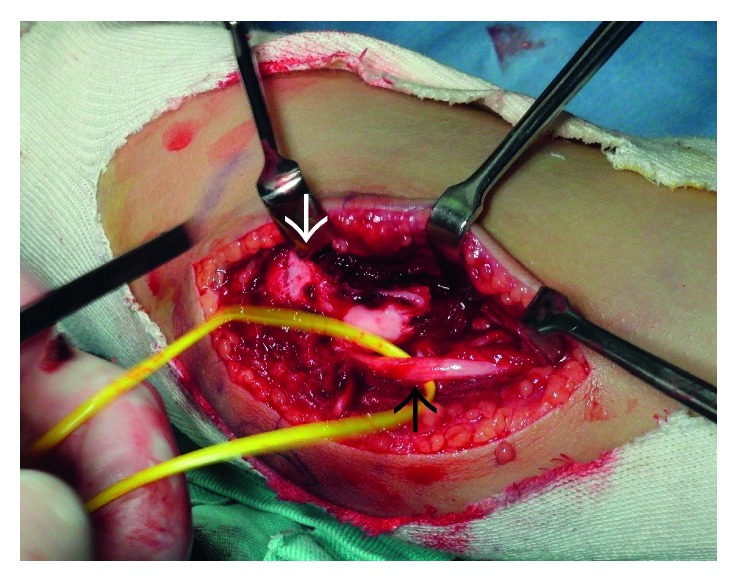
Intraoperative imaging. The white arrow shows the bone fragment, and the black arrow shows the ulnar nerve.

**Figure 3:**
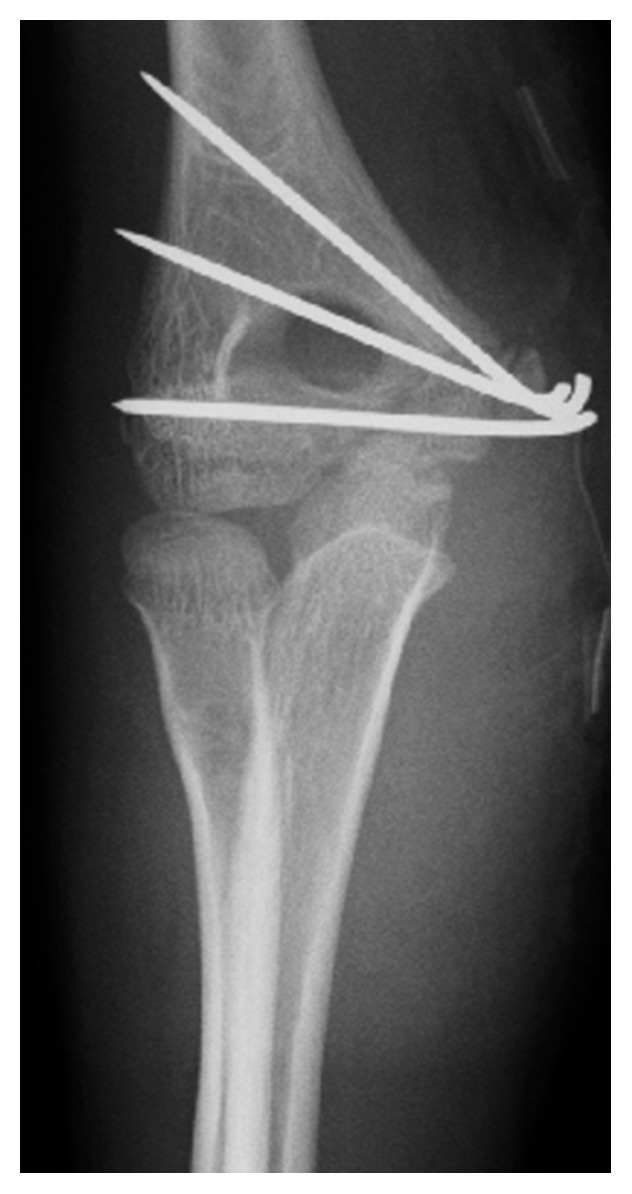
Postoperative anteroposterior radiography. The bone fragment was fixed in an accurate position with three smooth Kirschner wires.

An 11-year-old boy was injured after falling to the floor and landing on his right hand. He complained of severe pain in his dominant right elbow. He came to our hospital on the same day. The initial examination showed severe swelling on the medial side of his right elbow and pain. The initial X-ray studies of the elbow showed a fracture of the medial condyle of humerus. The bone fragment was displaced laterally by the traction of the flexor muscles; however, dislocation of the humeroulnar joint was not observed (Figure 1). We diagnosed the patient with a medial condyle fracture of the humerus (Salter–Harris [8] type IV, Milch type [9] I, and Kilfoyle type [5] III). On the next day, open reduction and internal fixation of the fracture were performed under general anesthesia. An incision was made over the fragment. First, the ulnar nerve was identified (Figure 2). The reduced bone fragment was fixed with three Kirschner wires in the accurate position. Osteosynthesis with a compression screw would have been the optimal treatment. Since multiple temporary fixations with wires were needed to fix the unstable bone fragment, we were afraid of bursting the fragment with a compression screw (Figure 3).

A long arm splint was applied from the upper arm to the metacarpophalangeal joints, with the forearm kept in a neutral position for four weeks.

**Figure 4:**
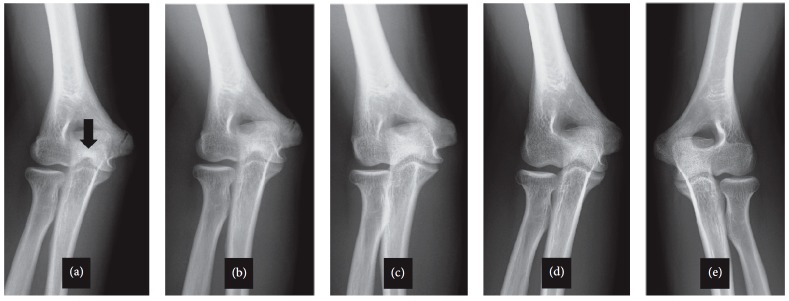
Follow-up from 1 to 4 years and contralateral anteroposterior radiography. (a) The 1-year follow-up examination. The black arrow shows the absorption of the trochlear groove (fishtail deformity). (b) The 2-year follow-up examination. (c) The 3-year follow-up examination. (d) The 4-year follow-up examination. (e) Contralateral anteroposterior radiography.

The three Kirschner wires were removed at 8 weeks after surgery under general anesthesia (Figure 4). Ulnar nerve palsy was not noted during the follow-up period. At 1 year after surgery, the absorption of the trochlear groove was observed on an anteroposterior view radiograph of the elbow and thus demonstrated a so-called “fishtail deformity.” However, the patient did not complain of elbow pain, and the range of motion was not limited.

The absorption of the trochlear groove was gradually remodeled before the most recent follow-up examination (at four years after surgery). At the latest follow-up examination, a good range of motion was observed, with 0 degrees of extension and 140 degrees of flexion.

The patient and the patient's parents were informed that this case study would be submitted for publication and provided their informed consent.

## 3. Discussion

Medial condyle fractures of the distal humerus are a very rare type of elbow injury [1–4]. The fracture line intersects the distal humerus and the medial metaphyseal-epicondylar segment [10]. Two mechanisms of fracture have been suggested. The first involves direct force to the apex of the flexed elbow as would occur in a fall which forces the olecranon into the medial condyle of the humerus [11]. The second proposed mechanism is an avulsion-type injury, which would be caused by stress to the medial collateral ligament and flexor insertions due to a fall onto the outstretched arm [1, 12].

Deformity of the trochlear groove after a lateral condyle fracture in children has been referred to as a “fishtail deformity.” Previous studies have reported that fishtail deformity was found in 40–60% of children after a lateral condyle fracture with ≥ 2 mm of displacement [13, 14]. Several authors described fishtail deformities and deformities of the trochlea after supracondylar and transcondylar fractures of the distal humerus [15–17]. The frequency of “fishtail deformity” after medal condyle fracture is unknown. It was previously suggested that “fishtail deformity” seldom causes functional or cosmetic problems, despite the abnormal radiographic appearance [18, 19]. However, one should note that an elbow with a fishtail deformity is mechanically weak and fragile, and care should be exercised to avoid damage to the trochlea at the initial treatment for a pediatric lateral condyle fracture [14]. There are some reports of distal humerus fracture due to the fragility caused by “fishtail deformity” [14, 20]. Moreover, long-term follow-up suggests that patients with fishtail deformity are prone to functional impairment, ongoing pain, and the development of early osteoarthrosis [21].

The blood supply to the trochlear epiphysis is important to the clinical outcome of medial or lateral condyle fractures of the elbow. Growth center involvement is another critical feature of pediatric medial humeral condyle fractures. The distal humerus is supplied by a solitary nutrient vessel [22]. Thus, vascular insult has a significant impact on healing and subsequent humeral development. Vascular supply to the trochlear epiphysis may be diminished, leading to the suppression of growth and/or fracture union, and results in cubitus varus deformity [23, 24]. Alternatively, growth may be stimulated at the injury site, yielding a cubitus valgus state. Osteonecrosis will occur if blood flow to the area ceases [24].

Thus, while there have been no reports on the topic, it is inferred that there are more cases of “fishtail deformity” after medial condyle fractures. As occurred in the current case, the improvement of ischemia of the trochlea after medial condyle fracture may be associated with the likelihood of recovery from transient “fishtail deformity”; however, the number of the patients of medial condyle fracture is too small to investigate this possibility.

In the current case, the varus deformity was not observed, and the “fishtail deformity” was transient. Although the “fishtail deformity” showed gradual improvement, it is unknown whether the healing process occurred due to accurate reduction and secure fixation, or whether it occurred due to the anatomical specificity of the vascular supply to the medial condyle.

## 4. Conclusions

We herein described a case of medial condyle fracture of the elbow with transient fishtail deformity after open reduction and internal fixation. The process by which ischemia of the trochlea improves is unknown. More case reports that include detailed follow-up examinations will be needed to further elucidate the pathophysiological characteristics of medial condyle fractures of the elbow.

## References

[1] Fowles J. V., Kassab M. T. (1980). Displaced fractures of the medial humeral condyle in children. *Journal of Bone & Joint Surgery, American Volume*.

[2] Chacha P. B. (1970). Fracture of the medial condyle of the humerus with rotational displacement: report of two cases. *Journal of Bone & Joint Surgery, American Volume*.

[3] Fahey J. J., O’Brien E. T. (1971). Fracture-separation of the medial humeral condyle in a child confused with fracture of the medial epicondyle. *Journal of Bone & Joint Surgery, American Volume*.

[4] Papavasiliou V., Nenopoulos S., Venturis T. (1987). Fracture of the medial condyle of the humerus in childhood. *Journal of Pediatric Orthopaedics*.

[5] Kilfoyle R. M. (1965). Fractures of the medial condyle and epicondyle of the elbow in children. *Clinical Orthopaedics and Related Research*.

[6] Hanspal R. S. (1985). Injury to the medial condyle in a child reviewed after 18 years. *Journal of Bone & Joint Surgery, British Volume*.

[7] Cothay D. M. (1967). Injury to the lower medial epiphysis of the humerus before development of the ossific centre. Report of a case. *Journal of Bone & Joint Surgery, British Volume*.

[8] Salter R. B., Harris W. R. (1963). Injuries involving the epiphyseal plate. *Journal of Bone & Joint Surgery, American Volume*.

[9] Milch H. (1964). Fracture and fracture dislocations of the humeral condyle. *Journal of Trauma: Injury, Infection, and Critical Care*.

[10] Lynsky M. L., Beebe A. (2012). When is a medial epicondyle fracture a medial condyle fracture?. *American Journal of Orthopedics*.

[11] Ghawabi M. H. (1975). Fracture of the medial condyle of the humerus. *Journal of Bone & Joint Surgery, American Volume*.

[12] Leet A. I., Young C., Hoffer M. M. (2002). Medial condyle fractures of the humerus in children. *Journal of Pediatric Orthopaedics*.

[13] Ippolito E., Tudisco C., Farsetti P., Caterini R. (1996). Fracture of the humeral condyles in children: 49 cases evaluated after 18-45 years. *Acta Orthopaedica Scandinavica*.

[14] Otsuka J., Horii E., Koh S., Hiroishi M. (2015). Unusual humeral medial condyle fracture in an adolescent because of a previous post-traumatic fishtail deformity: a case report. *Journal of Pediatric Orthopaedics B*.

[15] Kim H. T., Song M. B., Conjares J. N., Yoo C. L. (2002). Trochlear deformity occurring after distal humeral fractures: magnetic resonance imaging and its natural progression. *Journal of Pediatric Orthopaedics*.

[16] Bronfen C. E., Geffard B., Mallet J. F. (2007). Dissolution of the trochlea after supracondylar fracture of the humerus in childhood: an analysis of six cases. *Journal of Pediatric Orthopaedics*.

[17] Schulte D. W., Ramseier L. E. (2009). Fishtail deformity as a result of a non-displaced supracondylar fracture of the humerus. *Acta Orthopaedica Belgica*.

[18] Jakob R., Fowles J. V., Rang M., Kassab M. T. (1975). Observations concerning fractures of the lateral humeral condyle in children. *Journal of Bone & Joint Surgery, British Volume*.

[19] Glotzbecker M. P., Bae D. S., Links A. C., Waters P. M. (2013). Fishtail deformity of the distal humerus: a report of 15 cases. *Journal of Pediatric Orthopaedics*.

[20] Namba J., Tsujimoto T., Temporin K., Yamamoto K. (2011). Medial condyle fracture of the distal humerus in an adolescent with pre-existing fishtail deformity. A case report. *Emergency Radiology*.

[21] Hayter C. L., Giuffre B. M., Hughes J. S. (2010). Pictorial review: ‘fishtail deformity’ of the elbow. *Journal of Medical Imaging and Radiation Oncology*.

[22] Kimball J. P., Glowczewskie F. I., Wright T. W. (2007). Intraosseous blood supply to the distal humerus. *Journal of Hand Surgery, American Volume*.

[23] Ring D., Jupiter J. B. (2000). Fractures of the distal humerus. *Orthopedic Clinics of North America*.

[24] Beaty J. H., Kasser J. R. (2006). The elbow: physeal fractures, apophyseal injuries of the distal humerus, osteonecrosis of the trochlea and T-condylar fractures. *Rockwood and Wilkins’ Fractures in Children*.

